# Multi-Trigger Thermo-Electro-Mechanical Soft Actuators under Large Deformations

**DOI:** 10.3390/polym12020489

**Published:** 2020-02-23

**Authors:** Ebrahim Yarali, Reza Noroozi, Armin Yousefi, Mahdi Bodaghi, Mostafa Baghani

**Affiliations:** 1 Department of Engineering, School of Science and Technology, Nottingham Trent University, Nottingham NG11 8NS, UK; ebrahim.me20@gmail.com (E.Y.); 2 School of Mechanical Engineering, College of Engineering, University of Tehran, P.O. Box 11155-4563 Tehran, Iran; reza.noroozi@ut.ac.ir (R.N.); yousefi.armin@ut.ac.ir (A.Y.); baghani@ut.ac.ir (M.B.)

**Keywords:** multi-trigger soft actuators, thermo-electro-hyperelastic materials, large bending, semi-analytical solution, finite element method (FEM)

## Abstract

Dielectric actuators (DEAs), because of their exceptional properties, are well-suited for soft actuators (or robotics) applications. This article studies a multi-stimuli thermo-dielectric-based soft actuator under large bending conditions. In order to determine the stress components and induced moment (or stretches), a nominal Helmholtz free energy density function with two types of hyperelastic models are employed. Non-linear electro-elasticity theory is adopted to derive the governing equations of the actuator. Total deformation gradient tensor is multiplicatively decomposed into electro-mechanical and thermal parts. The problem is solved using the second-order Runge-Kutta method. Then, the numerical results under thermo-mechanical loadings are validated against the finite element method (FEM) outcomes by developing a user-defined subroutine, UHYPER in a commercial FEM software. The effect of electric field and thermal stimulus are investigated on the mean radius of curvature and stresses distribution of the actuator. Results reveal that in the presence of electric field, the required moment to actuate the actuator is smaller. Finally, due to simplicity and accuracy of the present boundary problem, the proposed thermally-electrically actuator is expected to be used in future studies and 4D printing of artificial thermo-dielectric-based beam muscles.

## 1. Introduction

Nowadays, soft robots, because of their capabilities involving movement and degrees of freedom, are attracting more and more scientific attention. They have several advantages over conventional robots. Soft robots can interact with humans and the environment more safely, and also could be employed in wearable devices. These robots are being developed to overcome the weakness of conventional robots in interacting with human and fragile biological objects [1,2,3,4]. Soft actuators are one of the critical components of soft robots. Thanks to the key characteristics of soft actuators (e.g., stimuli-sensitive, bio-compatible, light-weight, soft, etc.), they are mainly used as grippers to manipulate objects and in biomedical rehabilitation assistance as artificial muscles [5]. These actuators can convert several forms of energy into motion. Indeed, soft actuators change their shapes in response to external stimuli [6,7]. Actuators can be triggered by various stimulus such as heat, light, magnetic field, pneumatic pressure, electric filed, pH, and so on [8,9]. Shape-memory alloys, fluidic elastomer actuators, shape memory polymers, dielectric actuators (DEAs), ionic polymer-metal composite, and electro-magnetorheological elastomer actuators are common types of smart materials which could be used in soft actuators [10,11,12,13,14]. In thermal actuators heat is transformed into mechanical work [15]. DEAs as a class of actuators can generate strains higher than 100% in response to an electric field [16]. Furthermore, DEAs due to their unique features such as light-weight, high energy density, low cost, silent operation, and compliance are well-suited for artificial muscle applications, underwater robots, flexible displays, as well as in dielectric elastomer oscillators [17,18,19,20,21]. Dielectric elastomer oscillators enable distributed, autonomous signal generation, that can be controlled in a wide range by external signals or mechanical stimuli [22]. Therefore, the design and analysis of soft actuators are vital.

Dielectric-based soft actuators have been investigated both experimentally and numerically. From a modeling point of view, few studies have been reported on modeling of multi-trigger thermo-dielectric-based soft actuators in the presence of a coupling between the thermal and dielectric effects [23,24,25]. Most of the previous studies have examined the electric or thermal loading, separately and mostly are focused on their hyperelastic response. Since the base material of thermo-dielectric-based soft actuators is chosen from rubber-like materials, thus, they could withstand large deformation.

Historically, many researchers have investigated the behavior of these actuators. Due to the viscoelastic properties of these materials, the elastic modulus of dielectric actuators changes during operation [26]. A number of researches have been conducted to model the hyperelastic behavior of both isotropic and anisotropic dielectric materials [16,18,27,28,29,30,31,32,33,34,35,36,37]. Several hyperelastic models are being considered for representing large deformation behavior of elastomers, such as Neo-Hookean, Mooney-Rivlin, Ogden, Yeoh, and others. These models use strain energy function to describe the elastic behavior of the material [38]. The hyperelastic models have been divided into two main categories: phenomenological models based on description of an observed material behavior, and mechanistic models derived from information about the underlying material structures [39]. There are some researches suggesting analytical and semi-analytical solutions for modeling of dielectrics [1,28,40,41,42]. Vatandoost et al., [43] presented a conceptual design to provide a practical approach to have a large torsional deformation for two types of tubular elastomers. The developed model is useful in design and fabrication of soft actuators and robots. Sigaeva and Czekanski [44] introduced a universal model describing plane strain bending of a multilayered sector of a cylindrical tube, which took into account residual stress as well as nanoscale effects. Reinforced dielectric tubular actuators also have been studied by He et al., [45] numerically under a non-uniform axisymmetric torsion. They applied Mooney-Rivlin model and an ideal dielectric model to account for the electric and elastic parts of the reinforced actuator, respectively. One of the commercial dielectric elastomers is VHB 4905™ (3M, Saint Paul, MN, USA) commonly used in soft actuators. The electric and mechanical properties of this dielectric has been examined numerically and experimentally by Mehnet et al., [46]. Using deformation dependent permittivity in a dielectric-based tube, Ogden and Dorfman [47] investigated the elastic response of a tube. Motivated by this idea, Zeng et al., [48], recently, employing the deformation dependent permittivity, studied the effect of parameters such as instability, loss of tension and dielectric breakdown of a circular actuator at various applied stretches.

Most of the soft actuators in the literature have been investigated under single-stimuli conditions, while, recently multi-responsive soft actuators have attracted a great deal of interest due to their high controllability upon to different stimulus [49]. In this regard, thermo-electro-responsive soft actuators under finite bending are studied in this work. Based on the multiplicative decomposition of deformation gradient tensor theory, the total deformation gradient tensor is divided into an electro-mechanical part and a thermal part. Also, according to the non-linear electro-mechanical continuum theory, the electric dependent stress part is determined by introducing a nominal Helmholtz free energy density function. For the hyperelastic part of the model, Mooney-Rivlin model as well as an exp-exp model are considered. Finally, employing a second-order Runge-Kutta algorithm the stress components and induced moment are identified through a semi-analytic approach. To verify the proposed formulation, the pure mechanical bending and thermo-mechanical loading cases are solved via FEM in ABAQUS. In order to implement the exp-exp model into ABAQUS, a user-defined subroutine written in FORTRAN called UHYPER is prepared.

This paper is organized as follows. In Section 2.1, constitutive equations for electro-hyperelastic elastomers are presented. Then a Helmholtz free energy density function based on the Mooney-Rivlin model and an exp-exp model is proposed. In Section 2.2, the electric constant and hyperelastic parameters of VHB 4910 in the present model are calibrated based on the existing experiments in the literature. In Section 2.3, as a boundary-value problem, a non-linear continuum framework for finite bending of an actuator is introduced. In Section 3, results and relevant discussions are presented for different conditions, where the results are validated under purely mechanical and themo-mechanical loadings. We finally present a summary and draw conclusions in Section 4.

## 2. Materials and Methods

### 2.1. Constitutive Equations for Electro-Hyperelastic Elastomers

In current configuration, **E, D** and **P** stand for electric field vector, electric induction or electric displacement vector and polarization density vector, respectively. For a condensed material, it can be expressed as:

(1) $$D=\varepsilon_{0}E+P$$

where, *ε*_0_ denotes electric permittivity of the free space. The simplified form of Equation (1) for an isotropic material is [50]:

(2) $$D=\varepsilon_{0}\varepsilon_{r}E;\;P=\frac{\varepsilon_{r}-1}{\varepsilon_{r}}D$$

in which *ε_r_* is dielectric constant (or elastomer specific relative permittivity). It is noted that at free current, free electric charge and quasi static condition, the Maxwell’s equation is recast as:

(3) curl(E)=0; div(D)=0

where curl and div are computed with respect to the current position. Also, for reference configuration, the current and reference electric variables are connected using the deformation gradient tensor **F** as follows:

(4) $$E^{l}=F^{T}E;\;D^{l}=F^{-1}D$$

Considering Equations (3) and (4), the Maxwell’s equation in the reference configuration (i.e., Lagrangian form) is written as:

(5) $$Curl\left(E^{l}\right)=0;\;Div\left(D^{l}\right)=0$$

Following [51,52] and based on the non-linear electro-elasticity theory, the total Cauchy stress tensor, **T**, followed by a Maxwell’s concept for an electro-elastic material, can be expressed as:

(6) $$T=S+P⊗E+\varepsilon_{0}\left[E⊗E-\frac{1}{2}\left(E.E\right)I\right]$$

in which **I** is the second-order identity tensor. In addition, the total Cauchy stress tensor **T,** is a combination of mechanical (**S**) polarization, and electrostatic Maxwell stress tensors. But this superposition of electric and mechanical stresses under large deformations can be inaccurate. To overcome this issue, some researchers such as Dorfmann and Ogden [51] and Kumar and Sarangi [53] introduced an amended nominal Helmholtz free energy density function in which the total stress can be determined through this amended free energy $\Omega =\Omega \left(F,E^{l}\right)$. The general form of this energy is expressed as:

(7) $$\Omega \left(F,E^{l}\right)=\rho \phi \left(F,E^{l}\right)-\frac{1}{2}\varepsilon_{0}E^{l}.\left(b^{-1}E^{l}\right)$$

where **b** is the left Cauchy-green deformation tensor. Unlike the superposition of stresses, it is preferred to apply superposition of the strain energy function. Thus, the general relation between the total stress tensor, electric induction vector and magnetic induction vector with strain energy function for an incompressible isotropic electro-elastic material may be written as:

(8) $$T=-pI+F\frac{\partial \Omega }{F},\;D^{l}=-\frac{\partial \Omega }{\partial E^{l}}$$

wherein *p* is a Lagrange multiplier associated with the incompressibility constraint. Since strain energy function is in terms of invariants (*I*_1_:*I*_6_), they can be defined as [53,54]:

(9) $$\begin{array}{c} I_{1}=\mathrm{tr}\left(b\right),\;I_{2}=\frac{1}{2}\left(\mathrm{tr}{\left(b\right)}^{2}-\mathrm{tr}\left(b^{2}\right)\right),\;I_{3}=\det\left(b\right)=1\\ I_{4}=\left(E^{l}⊗E^{l}\right):I,\;I_{5}=\left(E^{l}⊗E^{l}\right):b^{-1}\\ I_{6}=\left(E^{l}⊗E^{l}\right):b^{-2} \end{array}$$

where, tr, ⊗ and : stand for trace, tensor product (or dyad) and double contraction, respectively. Therefore, the explicit form of **T** can be expressed as [53,54]:

(10) $$T=-pI+2\Omega_{1}b+2\Omega_{2}\left(I_{1}b-b^{2}\right)-2\Omega_{5}E⊗E-2\Omega_{6}\left(b^{-1}E⊗E+E⊗b^{-1}E\right)$$

In addition, the dielectric displacement vector in the current configuration can be derived as:

(11) $$D=-2\left(\Omega_{4}b+\Omega_{5}I+\Omega_{6}b^{-1}\right)E$$

in which, $\Omega_{i}\left(i=1:6\right)=\partial \Omega /\partial I_{i}$.

Finally, based on the work of Kumar and Sarangi [53] and adapting Mooney-Rivlin model [55] for hyperelastic part of the model, the complete form of the free energy density ($\Omega^{M.R}$) for isotropic electro-hyperelastic materials is computed as:

(12) $$\Omega^{M.R}=C_{1}\left(I_{1}-3\right)+C_{2}\left(I_{2}-3\right)+\frac{\varepsilon_{0}}{2}\left(C_{3}I_{4}+C_{4}I_{5}+C_{5}I_{6}\right)$$

Similarity, the complete form of the free energy density ($\Omega^{E.E}$) for isotropic electro-hyperelastic materials based on the exp-exp model [56] can be obtained as:

(13) $$\Omega^{E.E}=A_{1}\left[\left(\exp\left(m_{1}\left(I_{1}-3\right)\right)-1\right)\right]+B_{1}\left[\left(\exp\left(n_{1}\left(I_{2}-3\right)\right)-1\right)\right]+\frac{\varepsilon_{0}}{2}\left(C_{3}I_{4}+C_{4}I_{5}+C_{5}I_{6}\right)$$

Now, using Equations (10)–(13) and specifying the deformation gradient tensor, the stress and electric displacement vector could be identified.

### 2.2. Material Model Calibration

**Figure 1:**
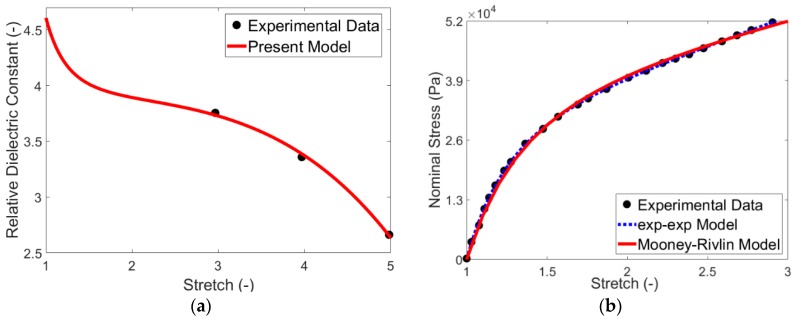
Experimental data [57] and the model prediction for VHB 4910 in two parts, (**a**) relativedielectric constant at frequency of 100Hz; (**b**) hyperelastic response at strain rate of 0.01 s.

To determine the electric parameters of the model, the experiments from Wissler and Mazza [57] for VHB 4910 acrylic-based dielectric elastomer are used. In this regard, they presented the deformation-dependent relative electric permittivity $\varepsilon_{r}=\varepsilon_{r}\left(\lambda \right)$ under equi-biaxial deformation loading as shown in Figure 1a. The deformation gradient **F** and electric field vector **E** for the experimental condition of Wissler and Mazza’ work [57] can be expressed as:

(14) $$F=\mathrm{diag}\left(\lambda ,\lambda ,\lambda^{-2}\right);\;E=\left(0,0,E_{0}\right)$$

Thus, considering Equations (11) and (12), the electric displacement vector is found as:

(15) $$D=\left(0,0,-E_{0}\varepsilon_{0}\left(\lambda^{-4}C_{3}+C_{4}+C_{5}\lambda^{4}\right)\right)$$

In addition, we have $D_{3}=\varepsilon_{0}\varepsilon_{r}E_{0}$. Therefore, the deformation dependent relative electric permittivity is obtained as:

(16) $$\varepsilon_{r}=-\left(C_{3}\lambda^{-4}+C_{4}+C_{5}\lambda^{4}\right)$$

Comparing Equation (16) and experimental data in Figure 1a, the dielectric parameters are identified as listed in Table 1. As one may observe from Figure 1a, the calibration of electric part of the model gives successful results compared to the experimental data.

In addition, to calibrate the hyperelatic part of the model, the experimental analysis done by Hossain et al. [58] for VHB 4910 acrylic-based dielectric elastomer is replicated here numerically. They provided many experiments on strain-dependent and time-dependent properties of VHB 4910. Samples were deformed in a pure homogenous and incompressible deformation as follows:

(17) $$F=\left[\begin{array}{ccc} \lambda^{-1/2} & 0 & 0\\ 0 & \lambda^{-1/2} & 0\\ 0 & 0 & \lambda \end{array}\right]$$

in which λ is longitudinal stretch in the direction *z*. The axial true stress tensor $\sigma^{HE}$ and nominal axial stress $P_{z}{}^{HE}$ can be calculated using strain energy density function $\Omega^{HE}$ as [59]:

(18) $$\begin{array}{c} \sigma^{HE}=2\frac{\partial \Omega^{HE}}{\partial I_{1}}(\lambda^{2}-\frac{1}{\lambda })+2\frac{\partial \Omega^{HE}}{\partial I_{2}}(\lambda -\frac{1}{\lambda^{2}});\\ P_{z}{}^{HE}=\frac{1}{\lambda }\sigma_{z}{}^{HE}=\frac{2}{\lambda }\frac{\partial \Omega^{HE}}{\partial I_{1}}(\lambda^{2}-\frac{1}{\lambda })+\frac{2}{\lambda }\frac{\partial \Omega^{HE}}{\partial I_{2}}(\lambda -\frac{1}{\lambda^{2}})=\frac{F}{A_{0}} \end{array}$$

For the hyperelastic behavior of the actuator, two strain energy functions namely Mooney-Rivlin $\Psi^{M.R}$ and exp-exp $\Psi^{E.E}$ are adapted. They can be expressed as:

(19) $$\begin{array}{c} \Psi^{M.R}=C_{1}\left(I_{1}-3\right)+C_{2}\left(I_{2}-3\right)\\ \Psi^{E.E}=A_{1}\left[\exp\left(m_{1}\left(I_{1}-3\right)\right)-1\right]+B_{1}\left[\exp\left(n_{1}\left(I_{2}-3\right)\right)-1\right] \end{array}$$

where, $C_{1},\;C_{2},\;A_{1},\;B_{1},\;m_{1},\;n_{1}$ are the material parameters. Considering Equations (18) and (19), the corresponding true and first Piola (nominal) stresses for the Mooney-Rivlin model are derived as:

(20) $$\begin{array}{l} \sigma^{M.R}=2\left(C_{2}+C_{1}\lambda^{-1}\right)\left(\lambda^{2}-\lambda^{-1}\right)\\ P_{z}{}^{M.R}=2\left(C_{2}+C_{1}\lambda^{-1}\right)\left(\lambda -\lambda^{-2}\right) \end{array}$$

Similarity, for the exp-exp model, in light of Equation (18), and Equation (19), the corresponding true and first Piola (nominal) stresses are calculated as:

(21) $$\begin{array}{l} \sigma^{E.E}=2\left(1-\frac{1}{\lambda }\right)A_{1}m_{1}\exp\left[m_{1}\left(\frac{2}{\lambda }+\lambda^{2}-3\right)\right]+2\left(\lambda -\lambda^{-2}\right)B_{1}n_{1}\exp\left[n_{1}\left(2\lambda +\frac{1}{\lambda^{2}}-3\right)\right]\\ P_{z}{}^{E.E}=2\left(\lambda -\lambda^{-2}\right)A_{1}m_{1}\exp\left[m_{1}\left(\frac{2}{\lambda }+\lambda^{2}-3\right)\right]+2\left(1-\lambda^{-3}\right)B_{1}n_{1}\exp\left[n_{1}\left(2\lambda +\frac{1}{\lambda^{2}}-3\right)\right] \end{array}$$

Finally, by performing a fitting procedure for Equations (20) and (21) and experimental data in Figure 1b, the hyperelastic parameters are obtained as reported in Table 1. As shown in Figure 1b, the calibration results for hyperelastic part of the model are in good agreement with those from experiments.

### 2.3. Non-Linear Continuum Framework of Finite Bending of the Actuator

**Figure 2:**
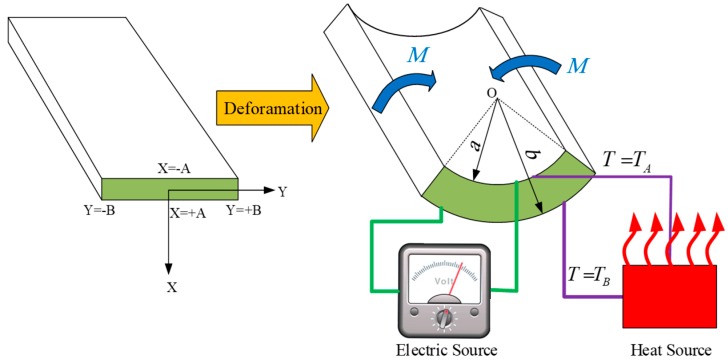
A schematic of plane-strain bending of a soft dielectric-based actuator under a thermo-electro-mechanical loading.

As mentioned previously, in this paper, as an application of thermally and electrically responsive actuators, large bending of a beam is examined. In this regard, an incompressible hyperelastic rectangular beam subjected to a plane-strain thermo-electro-mechanical bending is considered as depicted in Figure 2. Coordinates (*X*, *Y*, *Z*) and (*r*, *θ*, *z*) are cartesian and cylindrical coordinate systems used in reference and current configurations, respectively. The relation between reference and current configurations for large bending of the actuator can be written as:

(22) $$-A<X<+A,\;-B<Y<+B,\;a<r<b,\;-\frac{B}{\rho }<\theta <+\frac{B}{\rho }$$

where

(23) $$r=f\left(X\right),\;\theta =\frac{Y}{\rho },\;z=Z$$

and ρ denotes the mean radius of curvature of the beam.

In the cylindrical coordinates, the deformation gradient tensor **F** is introduced as:

(24) $$F=\left[\begin{array}{ccc} \frac{\partial r}{\partial X} & \frac{\partial r}{\partial Y} & \frac{\partial r}{\partial Z}\\ r\frac{\partial \theta }{\partial X} & r\frac{\partial \theta }{\partial Y} & r\frac{\partial \theta }{\partial Z}\\ \frac{\partial z}{\partial X} & \frac{\partial z}{\partial Y} & \frac{\partial z}{\partial Z} \end{array}\right]=\left[\begin{array}{ccc} \frac{\partial r\left(X\right)}{\partial X} & 0 & 0\\ 0 & \frac{r}{\rho } & 0\\ 0 & 0 & 1 \end{array}\right]=\mathrm{diag}\left(\frac{\partial r\left(X\right)}{\partial X},\frac{r}{\rho },1\right)$$

where X is the position vector in the reference coordinates (*X*, *Y*, *Z*). For a thermo-elastic analysis, the total deformation gradient is F(X,T), where the total volume ratio is J(X,T)=det F(X,T)>0. The multiplicative decomposition separates the total thermo-electro-elastic deformation gradient into an electro-mechanical contribution $F_{EM}$ and a purely thermal one $F_{T}$ as described in Equation (25), which represents the Duhamel-Neumann hypothesis in the nonlinear deformation theory [59].

(25) $$F=F_{T}F_{EM},\;J=J_{T}J_{EM}$$

where $J_{EM}=\det F_{EM}\;>0$ and $J_{T}=\det F_{T}\;>0$.

If the thermo-electric-elastic material is thermally isotropic, considering a non-isothermal deformation process, for a mechanically incompressible isotropic material, the deformation gradient $F_{T}$ may be given by an isotropic tensor according to [59] as:

(26) $$F_{T}=F(T)I\;F(T)=\exp\left[\int_{\theta_{0}}^{\theta }\alpha (\hat{T})\;d\hat{T}\right]>0$$

where ***F***(*T*) is a scalar-valued scalar function determining the thermal volume change, and α=α(T) stands for the temperature-dependent thermal expansion coefficient. For an isothermal process with no mechanical volume change (i.e., the material is mechanically incompressible), we have $J_{EM}=1$. Then, considering $\alpha_{0}$ as the linear thermal expansion coefficient and $T_{0}$ as the reference temperature, from Equation (27), one may find an approximate solution [59] as:

(27) $$F(T)\;=\exp\left[\int_{\theta_{0}}^{\theta }\alpha (\hat{T})\;d\hat{T}\right]=\exp(\alpha_{0}(T-T_{0}))≅1+\alpha_{0}(T-T_{0})$$

The steady state heat-conduction equation in absence of any heat source is written as:

(28) $$\frac{\partial^{2}T}{\partial X^{2}}+\frac{\partial^{2}T}{\partial Y^{2}}+\frac{\partial^{2}T}{\partial Z^{2}}=0$$

Considering the following Dirichlet boundary conditions:

(29) $$T\left(X=+A\right)=T_{B},\;T\left(X=-A\right)=T_{A}$$

the temperature distribution in the reference coordinate can be derived as:

(30) $$T=\left(\frac{\Delta T}{2A}\right)X+\frac{T_{B}+T_{A}}{2};\;\Delta T=T_{B}-T_{A}$$

in which ΔT is the temperature difference between lower and upper sides of the beam. Considering Equations (27) and (30), this yields:

(31) $$F_{T}=\left[1+\alpha_{0}\left(\left(\frac{T_{B}-T_{A}}{2A}\right)X+\frac{T_{B}+T_{A}}{2}-T_{0}\right)\right]I$$

Regarding Equations (25) and (31), one may argue that $F_{EM}=FF_{T}^{-1}$; therefore:

(32) $$F_{EM}={\left[1+\alpha_{0}\left(\left(\frac{T_{B}-T_{A}}{2A}\right)X+\frac{T_{B}+T_{A}}{2}-T_{0}\right)\right]}^{-1}\mathrm{diag}\left(\frac{\partial r\left(X\right)}{\partial X},\frac{r}{\rho },1\right)$$

According to $J_{EM}=1$, we have $J=J_{T}J_{EM}=J_{T}$. one obtains:

(33) $$\frac{\partial r}{\partial X}\frac{r}{\rho }≅\left[1+3\alpha_{0}\left(\left(\frac{T_{B}-T_{A}}{2A}\right)X+\frac{T_{B}+T_{A}}{2}-T_{0}\right)\right]V$$

In Equation (34), if we use direct integration, the relation between *r* and *X* is found as:

(34) $$r=\sqrt{2\rho X\left\{1+3\alpha \left(\frac{T_{B}-T_{A}}{4A}X+\frac{T_{B}+T_{A}}{2}-T_{0}\right)\right\}-2\rho A\left\{3\alpha \left(T_{0}-\frac{T_{B}+3T_{A}}{4}\right)-1\right\}+a^{2}}$$

The corresponding left Cauchy–Green deformation tensor $B_{EM}=F_{EM}F_{EM}^{T}$ is then recast as:

(35) $$B_{EM}={\left(1+\alpha \left(\frac{1}{2}\frac{\left(T_{B}-T_{A}\right)X}{A}+\frac{1}{2}T_{B}+\frac{1}{2}T_{A}-T_{0}\right)\right)}^{2}.\mathrm{diag}\left({\left(\frac{\partial r\left(X\right)}{\partial X}\right)}^{2},{\left(\frac{r\left(X\right)}{\rho }\right)}^{2},1\right)$$

Also, the quasi-static equilibrium equation in the radial direction in absence of body forces (in the current configuration) is expressed as:

(36) $$\frac{\partial \sigma_{rr}}{\partial r}+\frac{\sigma_{rr}-\sigma_{\theta \theta }}{r}=0$$

Taking into account the incompressibility condition, and integrating Equation (36) with respect to *r*, the following equation is found:

(37) $$\int_{a}^{r}\sigma_{rr}=\int_{a}^{r}\frac{\sigma_{\theta \theta }-\sigma_{rr}}{r}dr\Rightarrow \sigma_{rr}\left(r\right)-\sigma_{rr}\left(a\right)=\int_{a}^{r}\frac{\sigma_{\theta \theta }-\sigma_{rr}}{r}dr$$

Accounting for the boundary condition at r=a($\sigma_{rr}=0$), we have

(38) $$\sigma_{rr}\left(r\right)=\int_{a}^{r}\frac{\sigma_{\theta \theta }-\sigma_{rr}}{r}dr$$

It is then needed to identify two parameters *a* and *b* as the current internal and external radii of the structure. By employing a second-order Runge-Kutta method for Equation (36) and considering corresponding boundary conditions, parameters of *p*, *a* and *b*, the stresses can be determined. This process has been carried out by solving a nonlinear set of algebraic equations. Moreover, the moment (M) can be calculated using the hoop stress as:

(39) $$M=\int_{a}^{b}\sigma_{\theta \theta }rdr$$

As mentioned before, the proposed boundary-value problem can potentially perform as an actuator or manipulator in bending situation. Also, for simplicity, the electric field is applied in one direction (here, radial direction only). In another word, it is assumed that the electrodes are connected at the upper and lower sides of the actuator (see Figure 2). Thus, the applied electric field vector E only varies in radial direction in this paper ($E=\left(E_{1},0,0\right)$). Finally, considering Equations (11) and (24), the radial electric induction under a radial electric field can be expressed as:

(40) $$\begin{array}{cc} D_{r}= & -C_{4}E_{1}\varepsilon_{0}-\frac{18AC_{3}E_{1}\varepsilon_{0}\rho^{3}\left(2A\rho {\left(1-3T_{0}\alpha +3T_{A}\alpha \right)}^{2}+3\left(T_{A}-T_{B}\right)\alpha \left(a^{2}-r^{2}\right)\right)}{r^{2}{\left(4A\rho +\sqrt{2A\rho \left(2A\rho {\left(1-3T_{0}\alpha +3T_{A}\alpha \right)}^{2}+3\left(T_{A}-T_{B}\right)\alpha \left(a^{2}-r^{2}\right)\right)}\right)}^{2}}-\\ & \frac{C_{5}E_{1}\varepsilon_{0}r^{2}{\left(4A\rho +\sqrt{2A\rho \left(2A\rho {\left(1-3T_{0}\alpha +3T_{A}\alpha \right)}^{2}+3\left(T_{A}-T_{B}\right)\alpha \left(a^{2}-r^{2}\right)\right)}\right)}^{2}}{18A\rho^{3}\left(2A\rho {\left(1-3T_{0}\alpha +3T_{A}\alpha \right)}^{2}+3\left(T_{A}-T_{B}\right)\alpha \left(a^{2}-r^{2}\right)\right)} \end{array}$$

Finally, all necessary parameters for this problem are listed in Table 1.

## 3. Results

### 3.1. Verification

In this section, firstly, under a zero electric field and thermal effects (i.e., purely mechanical loading), the problem is verified using finite element software ABAQUS. Then, under coupling between thermal and mechanical loading, the proposed solution is verified. A moment is applied to one side of the actuator, while at the same time, a temperature difference is applied to the lower and upper surfaces of the actuator (as shown in Figure 2). It is noted that mesh type CPE8RHT, with an 8-node biquadratic displacement, bilinear temperature, reduced integration, hybrid with linear pressure was chosen for simulation. Also, the thickness of the actuator (2A) is set 0.2 m.

#### 3.1.1. Purely Mechanical Deformation

**Figure 3:**
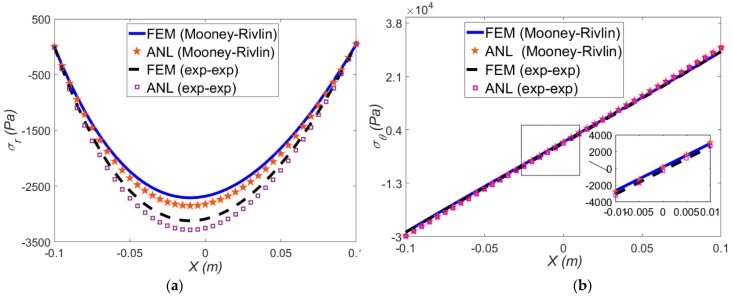
Variation of analytical and finite element method (FEM) (**a**) radial; (**b**) hoop stresses through the beam thickness with *ρ* = 0.5 m for two hyperelastic models. Analytical (ANL) stands for semi-analytical solution results.

In this section, the large bending of the beam under purely mechanical loading (i.e., external mechanical moment) is investigated through FEM and the developed semi-analytical method. At mean radius of curvature 0.5 m, the radial and hoop stress for both hyperelastic models are depicted in Figure 3a,b, respectively.

The comparison between the FEM and analytical method results for each strain energy functions reveals a good correspondence. Since the value of radial stress is small, a significant difference between two hyperelastic models can be observed. Under a fixed mean radius of curvature, the exp-exp model compared to Mooney-Rivlin model, predicts higher radial stresses. In other words, the exp-exp model gives more conservative results than Mooney-Rivlin model. In addition, the value of hoop stress for both strain energy functions are close (see Figure 3b). Meanwhile, under such large deformation (*ρ* = 0.5 m), in radial stress, an asymmetric response arising from the nonlinearity of the deformation is observed.

#### 3.1.2. Thermo-Mechanical Deformation

**Figure 4:**
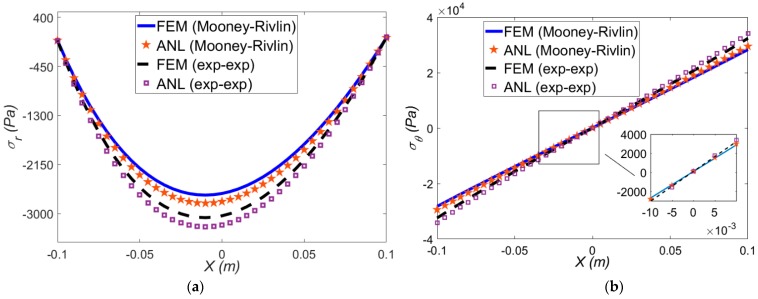
Variation of analytical and FEM (**a**) radial; (**b**) hoop stress through the beam thickness under *ρ* = 0.5 m and Δ*T* = 50 °C for two types of strain energy functions.

In this section, the large bending of the beam under thermo-mechanical loadings is examined through FEM and the proposed formulation. At a constant temperature difference of Δ*T* = 50 °C and *ρ* = 0.5 m, the radial and hoop stresses for both strain energies are depicted in Figure 4a,b, respectively.

As shown in Figure 4., the FEM and analytical results are in good agreement for both the Mooney-Rivlin and exp-exp modesl. The radial stresses in pure mechanical (Figure 3a) and thermo-mechanical (Figure 4a) deformation are different, so that the stresses in the thermo-mechanical deformation is lower than the pure mechanical loading, which means the applied temperature gradient intensifies the bending of the actuator. In addition, under a temperature difference at a fixed mean radius of curvature, the hoop stress (Figure 4b) compared to Figure 3b where there is no thermal loading has a lower value. Due to the thermal effect, a higher differences is observed between two hyperelastic models predictions. It is noted that, in order to keep brief, since the exp-exp and Mooney-Rivlin models predict almost similar trends, in each section only one of them is considered.

### 3.2. Effect of Temperature Difference in the Absence of Electric Field

**Figure 5:**
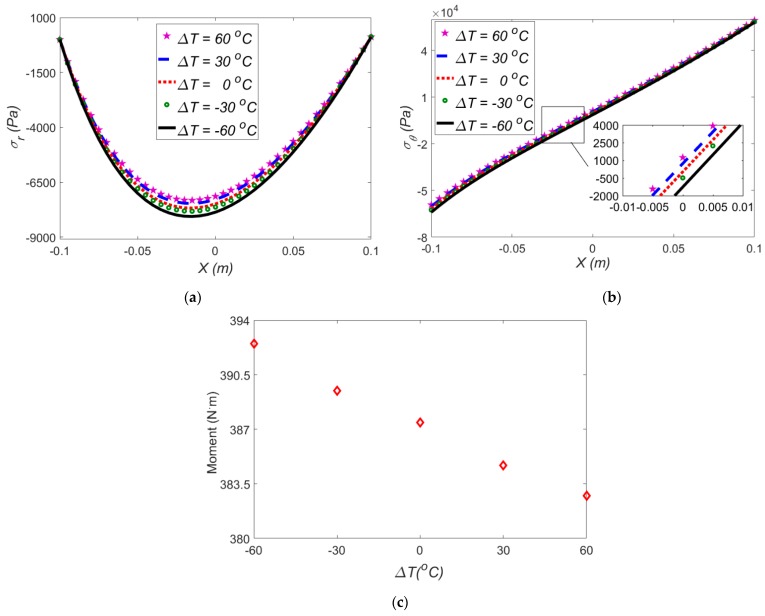
The effect of temperature gradients on (**a**) radial stress; (**b**) hoop stress; (**c**) induced moment for Mooney-Rivlin model in the absence of electric field at *ρ* = 0.3 m.

In this section, in the absence of an electric field, the effect of temperature gradients on the performance of the dielectric-based actuator under a large bending is examined especially on the induced moment and stress components. By adopting Mooney-Rivlin model for *ρ* = 0.3 m, in different temperature gradients, the radial, hoop stresses and induced moment are illustrated in Figure 5a–c.

As shown in Figure 5a,b, by changing the temperature difference from a high positive value to a high negative value, the radial and hoop stresses, and the induced moment start to increase. It is shown that, in Δ*T* = −60 °C, the radial stress is larger. It is noted that for a negative temperature gradient, an opposite thermal moment is produced; thus, an extra mechanical moment should be imposed to the beam to compensate this thermal moment. As shown in Figure 5b, the maximum hoop stress occurs at the lower surface of the beam in Δ*T* = −60 °C.

### 3.3. Effect of an Electric Field in the Absence of Temperature Difference

**Figure 6:**
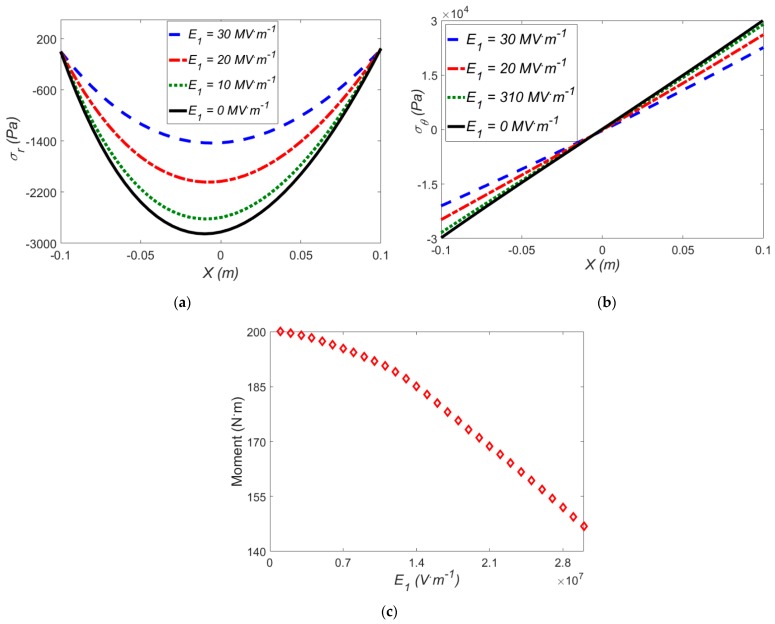
The effect of the electric field on (**a**) radial stress; (**b**) hoop stress; (**c**) induced moment for Mooney-Rivlin model with *ρ* = 0.5 m in the absence of temperature differences.

In this section, in the absence of thermal loading (i.e., electro-mechanical loading), the effect of the electric field on the actuator response at finite bending is examined. In this regard, the effect of uni-axial electric filed $E=\left(E_{1},0,0\right)$ on the stress components and induced moment for Mooney-Rivlin model at *ρ* = 0.5 m is reported in Figure 6.

As shown in Figure 6a,b, with increasing the electric field in a fixed mean radius of curvature *ρ* = 0.5 m, the amount of radial and hoop stresses are reduced, leading to a more symmetric form. Similar to the reasoning given for the effect of temperature gradients, under larger electric fields, an electric-induced moment is generated which means a lower mechanical moment is required. Consequently, lower hoop and radial stresses are developed in the structure. From Figure 6, it is noteworthy to mention that the stress components of the soft actuator can be controlled by altering the electric field. The effect of the electric field on the induced moment is depicted in Figure 6c, where at a higher electric field, we need a smaller moment. As indicated from Figure 5c and Figure 6c, the electric field has a more significant impact on the moment than the temperature difference. Therefore, in control of bending in soft actuators, the electric field sounds to be more promising.

#### The Effect of Applied Mean Radius of Curvature on the Stress Components and Induced Moment

**Figure 7:**
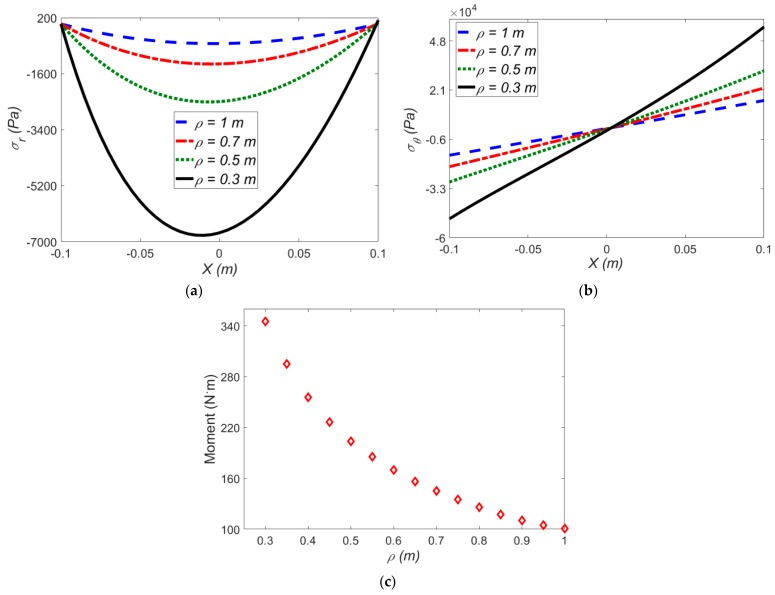
The effect of applied mean radius of curvature on (**a**) radial stress; (**b**) hoop stress; (**c**) induced moment for the exp-exp model at *E*_1_ = 20 MV·m^−1^ and Δ*T* = 0 °C.

Figure 7 reports the effect of applied mean radius of curvature on the stress components and induced moment for exp-exp hyperelastic model at a constant electric field (*E*_1_ =20 MV·m^−1^).

As shown in Figure 7a,b, the radial and hoop stresses decrease at smaller amounts of *ρ* in the presence of an electric field. To be more specific, from *ρ* = 0.3 to *ρ* = 0.5 m, the radial stress in the presence of electric field is reduced by 62%, while the radial stress in the absence of an electric field is lowered by 72%. Figure 7c shows the effect of mean radius of curvature on the induced moment.

### 3.4. Thermo-Electro-Mechanical Loading on the Actuator

**Figure 8:**
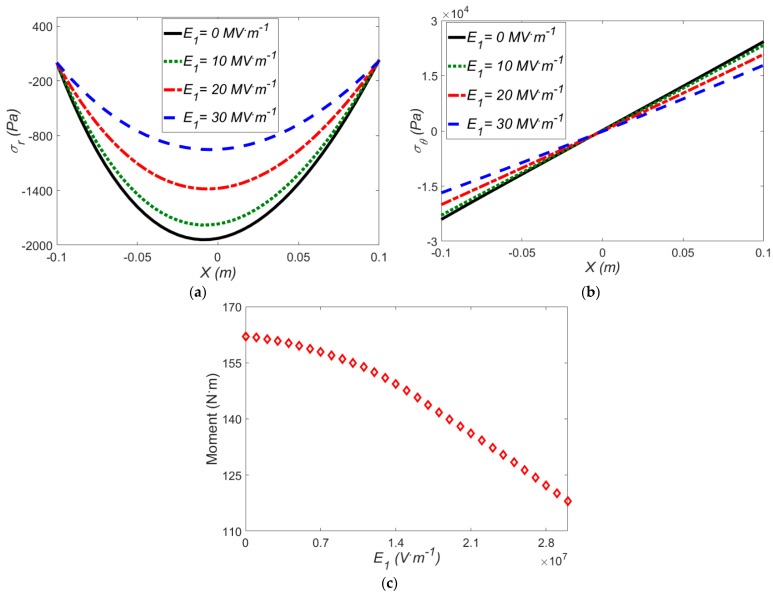
The effect of electric field on (**a**) radial stress; (**b**) hoop stress; (**c**) induced moment for Mooney-Rivlin model with *ρ* = 0.5 m and Δ*T* = 40 °C.

In this section, the response of a soft structure under thermo-electro-mechanical loading at large deformation is examined. The effects of uniform radial electric field on the radial stress, hoop stress and induced moment at a fixed specified mean radius of curvature for Mooney-Rivlin strain energy for Δ*T* = 40 °C, are shown in Figure 8.

Like in Figure 6, in Figure 8a, the radial stress grows with lowering the electric field; likewise, the radial stress curve becomes more asymmetric. Furthermore, the effect of temperature gradients is entirely in line with the electric field. Indeed, at higher temperature gradients, the amount of radial stress is reduced. Figure 8b illustrates that the maximum hoop stress occurs in the upper and lower sides of the beam in the absence of an electric field. As illustrated in Figure 8c, increasing the electric field results in a smaller induced moment. It is noted that the induced moment is less sensitive to the electric field in smaller values of the electric field.

#### 3.4.1. Effect of Applied Mean Radius of Curvature on the Stress and Induced Moment

**Figure 9:**
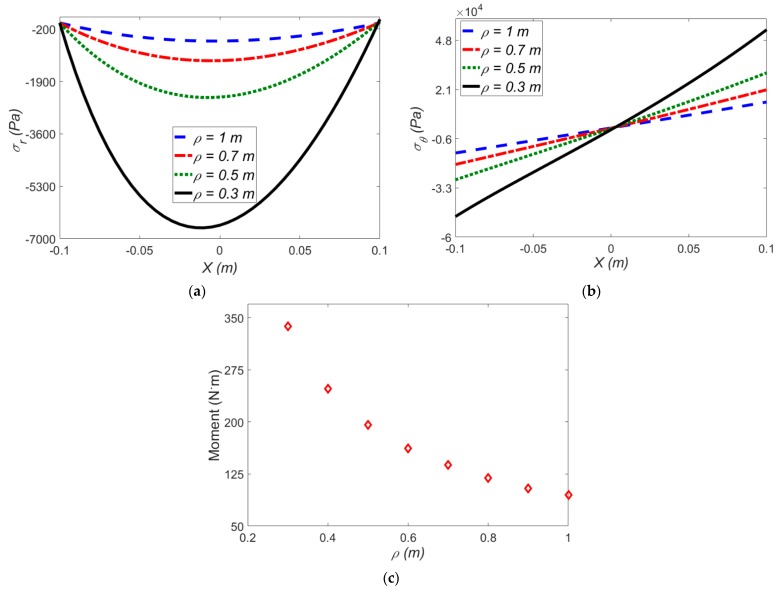
The effect of applied mean radius of curvature on (**a**) radial stress; (**b**) hoop stress; (**c**) induced moment for exp-exp model with *E*_1_ = 20 MV·m^−1^ and Δ*T* = 40 °C.

In this section, the effect of applied mean radius of curvature on the stress components and induced moment are investigated for exp-exp model at *E*_1_ = 20 MV.m^−1^ and Δ*T* = 40 °C and the relevant results are illustrated in Figure 9.

As depicted in Figure 9a, increasing the mean radius of curvature results in a smaller radial stress making the radial stress curve more symmetric. Figure 9b illustrates the hoop stress distribution. For a positive temperature gradient, as shown in Figure 9c, increasing *ρ* leads to a smaller induced moment.

#### 3.4.2. Effect of Electric Field and Temperature Gradient on the Electric Induction

**Figure 10:**
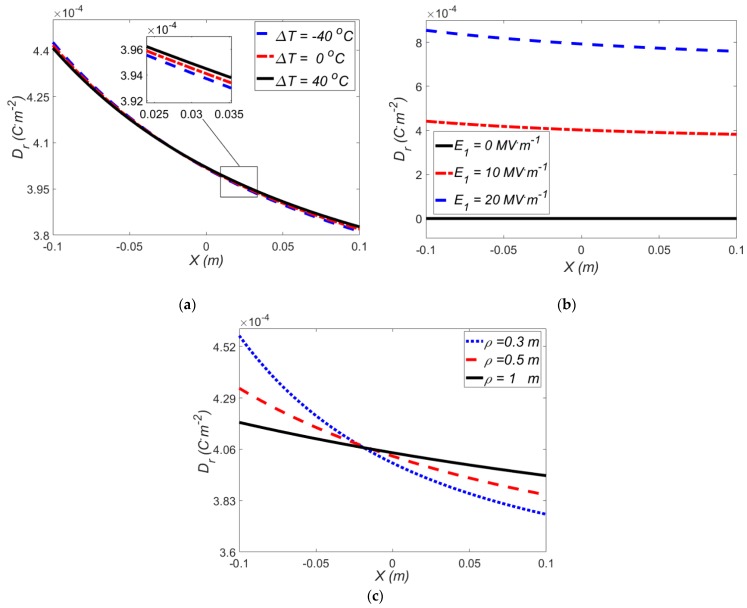
Variations of electric induction for exp-exp model with different (**a**) temperature gradients at *E*_1_ = 10 MV·m^−1^*, ρ* = 0.4 m; (**b**) electric fields at Δ*T* = 0 °C, *ρ* = 0.4 m; (**c**) applied mean radius of curvature at Δ*T* = 0 °C and *E*_1_ = 10 MV·m^−1^.

In this section, the effect of electric field and temperature gradients on the electric induction for exp-exp model is studied in Figure 10.

The effect of temperature gradient at a fixed electric field *E*_1_ = 10 MV·m^−1^ on the electric induction is depicted in Figure 10a, where a negative temperature gradient produces a higher radial electric field (at X < 0). A negative temperature gradient generates an opposite thermal moment and therefore larger stresses, induced moment and radial electric field are produced. It is observed that temperature gradient does not play a significant role on the electric induction in actuator. This is due to the value of the thermal expansion coefficient of VHB 4910. It means, if a dielectric with higher thermal expansion coefficient were chosen, the thermal part could play more prominent role in the results. Figure 10b depicts the effect of electric field on the electric induction at Δ*T* = 0 °C and *ρ* = 0.4 m, where a higher electric field results in a higher electric induction. Furthermore, the variation of the electric induction along the beam thickness is almost linear. Figure 10c shows the variation of electric induction with applied mean radius of curvature for Δ*T* = 0 °C and *E*_1_ = 10 MV·m^−1^. In addition, the variation of radial electric displacement in different mean radius of curvature is illustrated in Figure 10c which for larger mean radius of curvature, the radial electric displacement changes slightly.

## 4. Conclusions

In this paper, a multi-stimuli thermo-dielectric-based soft actuator under large bending was investigated. In this regard, the total deformation gradient tensor was calculated by decomposing two entirely separate parts: an electro-mechanical part and a thermal part. Firstly, a nominal Helmholtz free energy density function was adopted to pinpoint the electric-elastic-dependent stress part. Two strain energy density functions, i.e., Mooney-Rivlin and exp-exp models were considered in order to represent the hyperelastic term of the model. Finally, the nonlinear differential equations of the actuator were derived analytically, and then solved by utilizing the Runge–Kutta second-order to evaluate the total stress components, applied moment and electric induction. The boundary value problem results were verified by employing the FEM method in two purely mechanical and thermo-mechanical loading conditions. In this regard, a user-defined subroutine was developed to implement the exp-exp model into ABAQUS. FEM results confirmed the semi-analytical solution predictions. The semi-analytical solution of the actuator results in following remarks:

(1) By increasing the temperature differences in both sides of the actuator, in the absence of electric field, the amount of radial stress, hoop stress and applied moment are reduced. Moreover, at higher mean radius of curvature, the quantity of both radial and hoop stress is decreased.
(2) Imposing a negative temperature gradient to the beam results in an opposite thermal moment which means a larger mechanical moment should be applied to bend the beam to a desired mean radius of curvature.
(3) Radial stress, hoop stress, and applied moment decrease, with increasing the electro field in the absence of temperature differences, also the radial stress curve becomes more symmetric.
(4) The radial stress decreases by 62% in the presence of a fixed electric field and absence of temperature difference where the applied strain varies from *ρ* = 0.3 m to *ρ* = 0.5 m, although in the absence of an electric field, the radial stress drop is 72%.
(5) The effect of electric field on the stress components and applied moment at specified temperature gradients shows that the effect of temperature gradient is entirely in line with the electric field which means at higher temperature gradients we have lower stresses.
(6) The results reveal that due to the thermal expansion of the VHB 4910, the temperature gradient does not have a significant effect on the electric induction, while by increasing the electric field, the amount of the electric induction increases.

## Figures and Tables

**Table 1:** The material parameters adopted for the electro-hyperelastic model.

**Electric Part**					
Permittivity (ε_0_)	*C*_3_	*C*_4_		*C*_5_	
8.85 × 10^−12^ As/Vm	−0.7279	−3.879		0.001993	
**Thermal Part**					
*T*_A_		*T*_0_		α_0_	
300 K		300 K		180× 10^−6^ 1/K	
**Hyperelastic Part**					
Mooney-Rivlin		exp-exp			
*C*_1_	*C*_2_	*A*_1_	B_1_	m_1_	n_1_
1.463× 10^+4^ Pa	4114 Pa	1.076× 10^+6^ Pa	8.11× 10^+4^ Pa	0.005156	0.1956

## References

[1] Lee C., Kim M., Kim Y.J., Hong N., Ryu S., Kim H.J., Kim S. (2017). Soft robot review. Int. J. Control Autom. Syst..

[2] Yeo J.C., Yap H.K., Xi W., Wang Z., Yeow C.H., Lim C.T. (2016). Flexible and stretchable strain sensing actuator for wearable soft robotic applications. Adv. Mater. Technol..

[3] Zolfagharian A., Kouzani A.Z., Khoo S.Y., Moghadam A.A.A., Gibson I., Kaynak A. (2016). Evolution of 3D printed soft actuators. Sens. Actuators A Physical..

[4] Xiang C., Guo J., Sun R., Hinitt A., Helps T., Taghavi M., Rossiter J. (2019). Electroactive textile actuators for breathability control and thermal regulation devices. Polymers.

[5] Lin P.-W., Liu C.-H. (2019). Bio-inspired soft proboscis actuator driven by dielectric elastomer fluid transducers. Polymers.

[6] Song J., Zhang Y. (2019). From two-dimensional to three-dimensional structures: A superior thermal-driven actuator with switchable deformation behavior. Chem. Eng. J..

[7] Jia K., Wang M., Lu T., Wang T. (2019). Linear control of multi-electrode dielectric elastomer actuator with a finite element model. Int. J. Mech. Sci..

[8] Sachyani Keneth E., Scalet G., Layani M., Tibi G., Degani A., Auricchio F., Magdassi S. (2019). Pre-programmed tri-layer electro-thermal actuators composed of shape memory polymer and carbon nanotubes. Soft Robot..

[9] Slesarenko V., Engelkemier S., Galich P.I., Vladimirsky D., Klein G., Rudykh S. (2018). Strategies to control performance of 3d-printed, cable-driven soft polymer actuators: From simple architectures to gripper prototype. Polymers.

[10] Boyraz P., Runge G., Raatz A. (2018). An overview of novel actuators for soft robotics. Actuators.

[11] Yarali E., Baniassadi M., Baghani M. (2019). Numerical homogenization of coiled carbon nanotube reinforced shape memory polymer nanocomposites. Smart Mater. Struct..

[12] Yarali E., Mohammadi A., Mafakheri S., Baghani M., Adibi H. (2019). Mathematical modeling and experimental evaluation of a prototype double-tube Magnetorheological damper. SN Appl. Sci..

[13] Bodaghi M., Noroozi R., Zolfagharian A., Fotouhi M., Norouzi S. (2019). 4D printing self-morphing structures. Materials.

[14] Almomani A., Hong W., Hong W., Montazami R. (2017). Influence of temperature on the electromechanical properties of ionic liquid-doped ionic polymer-metal composite actuators. Polymers.

[15] Xia Q., Xia L., Shi T. (2018). Topology optimization of thermal actuator and its support using the level set based multiple–type boundary method and sensitivity analysis based on constrained variational principle. Struct. Multidiscip. Optim..

[16] He L., Lou J., Du J., Wang J. (2017). Finite bending of a dielectric elastomer actuator and pre-stretch effects. Int. J. Mech. Sci..

[17] Gupta U., Qin L., Wang Y., Godaba H., Zhu J. (2019). Soft robots based on dielectric elastomer actuators: A review. Smart Mater. Struct..

[18] Kadooka K., Taya M., Naito K., Saito M. Modeling of a corrugated dielectric elastomer actuator for artificial muscle applications. Proceedings of the Electroactive Polymer Actuators and Devices (EAPAD).

[19] Qin L., Cao J., Tang Y., Zhu J. (2018). Soft freestanding planar artificial muscle based on dielectric elastomer actuator. J. Appl. Mech..

[20] Alibakhshi A., Heidari H. (2019). Analytical approximation solutions of a dielectric elastomer balloon using the multiple scales method. Eur. J. Mech. A Solids.

[21] Zhang Q., Zhang Z., Xu N., Yang H. (2020). Dielectric properties of P(VDF-TrFE-CTFE) composites filled with surface-coated TiO2 nanowires by SnO2 nanoparticles. Polymers.

[22] Henke E.M., Wilson K.E., Anderson I. (2018). Modeling of dielectric elastomer oscillators for soft biomimetic applications. Bioinspir. Biomim..

[23] Wu T.-H., Li X.-Y. (2019). Elliptical crack problem in magneto-electro-thermo-elasticity of transversely isotropic materials: 3D analytical and numerical solutions. Int. J. Eng. Sci..

[24] Mehnert M., Hossain M., Steinmann P. (2018). Numerical modeling of thermo-electro-viscoelasticity with field-dependent material parameters. Int. J. Non Linear Mech..

[25] Ghobadi A., Beni Y.T., Golestanian H. (2019). Size dependent thermo-electro-mechanical nonlinear bending analysis of flexoelectric nano-plate in the presence of magnetic field. Int. J. Mech. Sci..

[26] Nguyen C.H., Alici G., Mutlu R. Modeling a soft robotic mechanism articulated with dielectric elastomer actuators. Proceedings of the 2014 IEEE/ASME International Conference on Advanced Intelligent Mechatronics.

[27] Goulbourne N.C. (2011). A constitutive model of polyacrylate interpenetrating polymer networks for dielectric elastomers. Int. J. Solids Struct..

[28] Li T., Keplinger C., Baumgartner R., Bauer S., Yang W., Suo Z. (2013). Giant voltage-induced deformation in dielectric elastomers near the verge of snap-through instability. J. Mech. Phys. Solids.

[29] Patrick L., Gabor K., Silvain M. (2007). Characterization of dielectric elastomer actuators based on a hyperelastic film model. Sens. Actuators A Physical..

[30] Siboni M.H., Castañeda P.P. (2019). Constitutive models for anisotropic dielectric elastomer composites: Finite deformation response and instabilities. Mech. Res. Commun..

[31] Su Y., Wu B., Chen W., Destrade M. (2019). Finite bending and pattern evolution of the associated instability for a dielectric elastomer slab. Int. J. Solids Struct..

[32] Volpini V., Bardella L., Gei M. (2019). A note on the solution of the electro-elastic boundary-value problem for rank-two laminates at finite strains. Meccanica.

[33] Wissler M., Mazza E. (2005). Modeling of a pre-strained circular actuator made of dielectric elastomers. Sens. Actuators A Physical..

[34] Siboni M.H., Castañeda P.P. (2014). Fiber-constrained, dielectric-elastomer composites: Finite-strain response and stability analysis. J. Mech. Phys. Solids.

[35] Siboni M.H., Castañeda P.P. (2019). Fiber-Constrained Dielectric Elastomer Composites: Finite Deformation Response and Instabilities Under Non-Aligned Loadings. Int. J. Solids Struct..

[36] Almasi A., Baghani M., Moallemi A. (2017). Thermomechanical analysis of hyperelastic thick-walled cylindrical pressure vessels, analytical solutions and FEM. Int. J. Mech. Sci..

[37] Noroozi R., Ataee A. (2018). Behavioral Optimization of Pseudo-Neutral Hole in Hyperelastic Membranes Using Functionally graded Cables. J. Comput. Appl. Mech..

[38] Garcia L.A., Trindade M.A. (2019). Finite element modeling and parametric analysis of a dielectric elastomer thin-walled cylindrical actuator. J. Braz. Soc. Mech. Sci. Eng..

[39] Moseley P., Florez J.M., Sonar H.A., Agarwal G., Curtin W., Paik J. (2016). Modeling, design, and development of soft pneumatic actuators with finite element method. Adv. Eng. Mater..

[40] Koh S.J.A., Li T., Zhou J., Zhao X., Hong W., Zhu J., Suo Z. (2011). Mechanisms of large actuation strain in dielectric elastomers. J. Polym. Sci. Part B Polym. Phys..

[41] Lu T., Huang J., Jordi C., Kovacs G., Huang R., Clarke D.R., Suo Z. (2012). Dielectric elastomer actuators under equal-biaxial forces, uniaxial forces, and uniaxial constraint of stiff fibers. Soft Matter.

[42] Zhao X., Suo Z. (2010). Theory of dielectric elastomers capable of giant deformation of actuation. Phys. Rev. Lett..

[43] Vatandoost H., Norouzi M., Alehashem S.M.S., Smoukov S.K. (2017). A novel phenomenological model for dynamic behavior of magnetorheological elastomers in tension–compression mode. Smart Mater. Struct..

[44] Sigaeva T., Czekanski A. (2018). Finite bending of a multilayered cylindrical nanosector with residual deformations. Math. Mech. Solids.

[45] He L., Lou J., Du J., Wu H. (2018). Voltage-driven nonuniform axisymmetric torsion of a tubular dielectric elastomer actuator reinforced with one family of inextensible fibers. Eur. J. Mech. A Solids.

[46] Mehnert M., Hossain M., Steinmann P. (2019). Experimental and numerical investigations of the electro-viscoelastic behavior of VHB 4905TM. Eur. J. Mech. A Solids.

[47] Dorfmann L., Ogden R.W. (2018). The effect of deformation dependent permittivity on the elastic response of a finitely deformed dielectric tube. Mech. Res. Commun..

[48] Zeng C., Gao X. (2019). Effect of the deformation dependent permittivity on the actuation of a pre-stretched circular dielectric actuator. Mech. Res. Commun..

[49] Li H., Go G., Ko S.Y., Park J.-O., Park S. (2016). Magnetic actuated pH-responsive hydrogel-based soft micro-robot for targeted drug delivery. Smart Mater. Struct..

[50] Dorfmann L., Ogden R.W. (2014). Nonlinear Theory of Electroelastic and Magnetoelastic Interactions.

[51] Dorfmann A., Ogden R. (2005). Nonlinear electroelasticity. Acta Mech..

[52] Kovetz A. (2000). Electromagnetic Theory.

[53] Kumar D., Sarangi S. (2019). Electro-magnetostriction under large deformation: Modeling with experimental validation. Mech. Mater..

[54] Kumar D., Sarangi S., Saxena P. (2020). Universal relations in coupled electro-magneto-elasticity. Mech. Mater..

[55] Mooney M. (1940). A theory of large elastic deformation. J. Appl. Phys..

[56] Mansouri M., Darijani H. (2014). Constitutive modeling of isotropic hyperelastic materials in an exponential framework using a self-contained approach. Int. J. Solids Struct..

[57] Wissler M., Mazza E. (2007). Electromechanical coupling in dielectric elastomer actuators. Sens. Actuators A Phys..

[58] Hossain M., Vu D.K., Steinmann P. (2012). Experimental study and numerical modelling of VHB 4910 polymer. Comput. Mater. Sci..

[59] Holzapfel G.A. (2002). Nonlinear solid mechanics: A continuum approach for engineering science. Meccanica.

